# Mechanisms of Anxiety Among Doctoral Students in China

**DOI:** 10.3390/bs15020105

**Published:** 2025-01-21

**Authors:** Fan Bai, Feng Zhang, Yeqi Xue

**Affiliations:** School of Education, Beijing Institute of Technology, Beijing 100081, China; 13001025985@163.com (F.B.); 13610683958@163.com (Y.X.)

**Keywords:** in-school doctoral students, anxiety, full-time, part-time, formation mechanisms

## Abstract

The anxiety of doctoral students in school has consistently been a major concern in society and the medical community, stemming from pressures, such as the pursuit of identity within highly professional and fiercely competitive academic communities, the urgent drive for high-level original scientific research output, and the need to break through the limits of personal ability to complete in-depth academic training. Current research has focused on the prevalence of, causes of, and intervention strategies for anxiety among doctoral students, but it still exhibits deficiencies, such as overly generalized analytical methods, neglect of the diversity within the doctoral student population, and the incomplete theoretical framework for the mechanisms of influence. Therefore, our study aims to examine the anxiety status of different categories of doctoral students and to explore their anxiety intervention mechanisms. Specifically, we employ a mixed research method combining quantitative and qualitative approaches to address issues, such as the influencing factors of anxiety in different types of doctoral students, the mechanisms of action of each factor, and the formation of an influence mechanism framework. The results indicate that anxiety is prevalent among doctoral students, with the key influencing factors being gender, age, love and marriage pressure, and self-perception. Full-time and part-time doctoral students exhibit different anxiety states and causes on account of varying pressures related to graduation, employment, and family factors. Our research distinguishes the heterogeneity of anxiety among different types of doctoral students in China, innovatively constructing a set of anxiety intervention mechanisms for doctoral students. It aims to provide policy insights for the adjustment of their anxiety and hopes to offer novel perspectives and exemplary references for the theoretical research and practical exploration of doctoral students’ anxiety in other countries around the world.

## 1. Introduction

Doctoral students, as significant contributors to academia, are central to the knowledge production and reproduction system in universities and form a robust force in driving technological innovation (Thune, 2009). The substantial responsibility for scientific progress that global doctoral students bear, coupled with the labor market changes arising from their steady numerical growth, subjects the in-school doctoral student population to immense pressure. This pressure manifests as challenges, such as poor academic performance and increased suicide risk, making the anxiety emerging under high stress a focal point of current academic attention. The psychological predicaments confronting doctoral candidates on campus are multifaceted.

Focusing on the psychological predicaments at the micro level, anxiety phenomena arise among doctoral students in school amidst the “publish or perish” culture, the advisor–advisee relationship, and the highly intensive academic tasks. Firstly, Larson et al. (2014) analyzed the ability of academia to absorb new doctoral graduates from a “birth rate” perspective, revealing that only 12.8% of doctoral graduates were able to obtain academic positions in the United States. The pressure to publish journal articles and academic manuscripts in order to secure employment is leading to a situation where young scholars worldwide are publishing incessantly and perishing; the embarrassing imperative “publish or perish” makes graduate students aware of competition among peers and the decrease in the number of open tenure track positions, leading to their anxiety (Alvarez et al., 2014; Vossen, 2017). Secondly, In a study conducted by Becerra et al. (2021) on the advisor–advisee relationship, results indicate that doctoral students who are satisfied with their advisor are less likely to frequently seek physical and mental well-being services at medical clinics and enjoy better sleep quality and superior mental health conditions. Among them, the probability of participants with the lowest 10% advisor satisfaction seeking medical attention due to mental health issues is expected to be 76%, while the probability of participants with the highest 10% advisor satisfaction is 41%. Communication barriers, mismatched expectations, and a lack of emotional care in the advisor advisory relationship are important factors that trigger doctoral students’ anxiety. Thirdly, Lonka et al. (2019) studied the writing profiles of doctoral students at the University of Helsinki, Finland, in which all participants emphasized the burdensome workload. Intensive research is often accompanied by sustained output requirements and high research uncertainty, which can exacerbate the anxiety of doctoral students.

**Figure 1:**
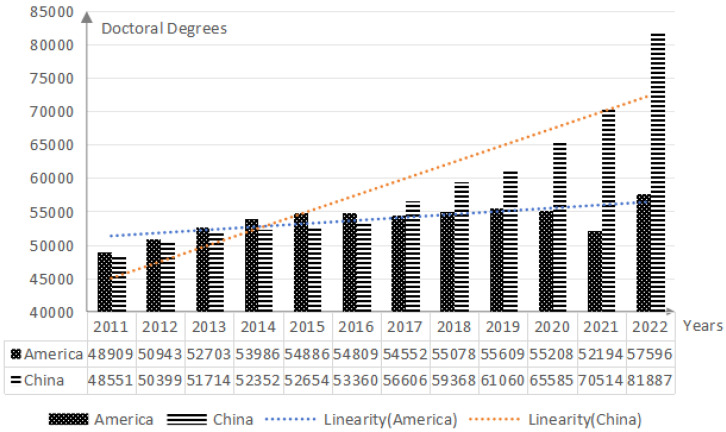
Number of doctoral degrees awarded in the United States and China from 2011 to 2022.^1^

Focusing on the psychological predicaments at the macro level, doctoral students in school experience anxiety in the employment environment characterized by the depreciation of academic qualifications and within the cultivation mechanism of strict standards. With the deepening development of the knowledge economy, global doctoral education is characterized by both scale and quality. In 2022, the number of doctoral degrees awarded by American universities was 57,596, with a year-over-year growth rate of 10.3% (NSF, 2023). Especially, in China, official higher education institutions have awarded 81,887 doctoral degrees, with a year-over-year growth rate of 16.13% (Figure 1) (Ministry of Education of the People’s Republic of China, 2022, 2023). Behind the increasing trend in the supply of doctoral candidates, there still lurks the persistent issue of the continuous rise in the rate of delayed completion among them. According to the algorithm of Yuan and Wang (2014), the calculation results of the delayed graduation rate of Chinese doctoral students from 2013 to 2022 are shown in Table 1. In the past decade, more than half of doctoral students have delayed their graduation, which means that the time for doctoral students to participate in academic research in school has been extended, which can easily lead to concerns about their future academic career development. The alteration in academic deadlines may not only result in doctoral students missing out on their original career development opportunities but may also have to bear unexpected tuition and living expenses, further increasing their financial pressure. These multiple factors together lead to enormous psychological pressure and self-identity crisis for doctoral students, breeding unpredictable anxiety emotions. Meanwhile, the expansion of the doctoral education scale has brought about a series of social competition issues. For example, the increase in doctoral graduates leads to an oversupply of highly qualified academic personnel, creating a structural imbalance in the labor market where job seekers far outnumber available tenure-track positions (Bekova & Dzhafarova, 2019; Golde & Dore, 2001). This mismatch and inefficiency in the doctoral graduate employment market result in degree devaluation and employment difficulties, heightening future development anxiety among doctoral students (Servage, 2009). Additionally, the expansion of the doctoral training system intensifies competition for resources and achievements in academia. Enhancing the quality of doctoral education is a core strategy for effectively addressing academic competition and cultivating high-level talent. Consequently, doctoral training standards have been continually raised, with increasingly stringent oversight mechanisms. Doctoral students are required to maintain a high level of academic focus and creativity amid frequent evaluations and the challenge of high-quality publications, leading to significant anxiety within this group (Kurtz-Costes et al., 2006; Liu et al., 2019).

**Table 1:** Delayed graduation rate of Chinese doctoral students from 2013 to 2022.^2^

Year	Actual Graduates/Person	Estimated Graduates/Person	Actual Graduation Rate (%)	Delayed Graduation Rate (%)
2013	53,139	139,411	38.12%	61.88%
2014	53,653	146,941	36.51%	63.49%
2015	53,778	149,190	36.05%	63.95%
2016	55,011	154,102	35.70%	64.30%
2017	58,032	161,799	35.87%	64.13%
2018	60,724	169,022	35.93%	64.07%
2019	62,578	172,824	36.21%	63.79%
2020	66,176	177,884	37.20%	62.80%
2021	72,019	189,744	37.96%	62.04%
2022	82,320	193,127	42.62%	57.38%

Therefore, to stand out in the fiercely competitive environment and achieve knowledge advancement, academic recognition, and career development, doctoral students face anxiety because of the instability in the outcomes of their struggle with real-world competition and self-expectations. Under the multiple pressures of learning, research, and career choice, 36% of respondents in a survey by Nature reported seeking help for anxiety or depression caused by their doctoral studies (Woolston, 2019). Given the current large doctoral student population, anxiety issues have transcended being a phenomenon and have increasingly emerged as a significant issue that demands urgent resolution and broad attention.

Anxiety is a psychological response to stress experienced by normal individuals (Huang, 2020). Each experience of anxiety includes the perception of danger, thoughts of harm, and physiological processes of alertness and activation (Moss, 2002). When these responses are prolonged or excessively intense, they can develop into anxiety disorders (Huang, 2020). Doctoral students often experience anxiety due to irrational expectations of their doctoral life and the resulting reality gap, commonly manifesting as persistent tension, low mood, and depression (Levecque et al., 2017). This can lead to a series of somatic symptoms, such as sleep problems due to worry, irritability, and physical discomfort (Garcia-Williams et al., 2014; Levecque et al., 2017). Additionally, social symptoms may emerge, including behavioral avoidance and reduced social functioning in interactions, personality disorders, stemming from the immediate anxiety related to academic, daily life, and employment issues faced by doctoral students, which have not yet developed into deep-seated anxiety disorders (Huang, 2020; Nogueira-Martins et al., 2004). Therefore, this study focuses on the psychological reactions of doctoral students in the context of individual development and social interaction. From a social psychology perspective, it examines the early anxiety symptoms induced by anxiety among in-school doctoral students, aiming to control the potential exacerbation of their anxiety and prevent the formation of pathological anxiety disorders in the later stage.

Specifically, the interplay of multiple factors contributes to the formation of anxiety among doctoral students, and this emotional state exhibits variability and diversity across doctoral candidates of different age groups, gender compositions, and academic disciplines. Firstly, in terms of academic, the main sources of anxiety for young doctoral students are the desire for academic advancement and the expectation of early career success, while the anxiety of older doctoral students mainly comes from time management and energy allocation issues under academic pressure and family responsibilities. Secondly, in terms of daily life, doctoral students in science and engineering may experience a lack of emotional support due to a relatively closed academic environment and social circle during long-term experimental work, while doctoral students in humanities and social sciences are more anxious about lower economic and employment security. Thirdly, in terms of employment, compared to male doctoral students, female doctoral students may face the dual psychological burden of workplace gender bias and family role expectations in future job hunting due to social questioning of their professional abilities, limitations on career development, and an excessive emphasis on family responsibilities. Fourthly, at the level of the interaction between academic and life factors, when doctoral students face obstacles in their research ideas, they may develop unhealthy habits, such as an irregular diet, lack of sleep, and lack of exercise due to an excessive investment in research, leading to decreased physical functions, scattered attention, and further hindering the progress of academic research. Fifthly, at the level of the interaction between academic and employment factors, the poor quantity and quality of academic achievements of doctoral students will directly weaken their competitiveness in the job market, and the uncertain employment prospects will also lead to a lack of motivation for doctoral students to conduct academic research. Sixthly, at the level of the interaction between life and employment factors, doctoral students with poor physical health are less likely to be favored by employers, while doctoral students are prone to negative emotions, such as irritability and anxiety under severe employment pressure. Therefore, this study focuses on the self-perceived anxiety symptoms of in-school doctoral students, aiming to provide early intervention for their anxiety, so as to help them better adapt to the pressures of daily life and preventing the evolution of long-term negative emotions into anxiety disorders.

The issue of anxiety among in-school doctoral students has garnered significant attention in academia. First, current research has demonstrated the prevalence and importance of anxiety among doctoral students and explored the series of social problems that it triggers. For instance, Evans et al. (2018a) surveyed 2279 graduate students (90% of whom were doctoral students) from various countries and regions, finding that graduate students are over six times more likely to experience depression and anxiety compared to the general population. Marais et al. (2018) focused on the French doctoral student population, finding that 42% of the sample exhibited abnormal anxiety levels. Doctoral students experience higher anxiety levels than other populations (Barry et al., 2018). When anxiety intensifies, symptoms of anxiety disorders can lead to suicidal ideation, causing harm to both physical and psychological health, while also inciting social panic (Engin et al., 2009). Suicide is the fourth leading cause of death among individuals aged 15–29, highlighting the urgent need to address anxiety among in-school doctoral students (World Health Organization [WHO], 2021). Second, existing research often relies on survey data to conduct statistical analyses on the anxiety of in-school doctoral students and explore its influencing mechanisms, yielding corresponding results. For example, Jones-White et al. (2022) used confirmatory factor analysis and binary logistic regression to demonstrate that a sense of belonging reduces the likelihood of doctoral students experiencing clinical anxiety and depression symptoms, while academic pressure, interpersonal pressure, and financial pressure increase this likelihood. Singh (2020) used a hierarchical linear regression model to show that age and research self-efficacy are negatively correlated with anxiety levels in doctoral students. Third, in response to the increasingly severe anxiety situation among doctoral students, current research has focused on intervention pathways. Waight and Giordano (2018) proposed non-academic support services to address psychological health, including anxiety, from five angles: identifying support service markers, providing online self-help resources, organizing specialized workshops, ensuring equal access to support, and enhancing advisor training. Byrom et al. (2022) emphasized providing more rigorous academic support to bolster doctoral students’ confidence, reduce their fear of failure, and enhance self-efficacy, thereby positively addressing issues of anxiety and depression.

However, these studies have the following limitations: first, many rely on psychological health measurement scales or survey data for analysis. This paradigm can only reveal the general causes of anxiety from a macro perspective, making it difficult to deeply explore the nature of individual anxiety among in-school doctoral students and provide detailed explanations and psychological interventions for individuals. Second, current research often views the doctoral student population as a homogeneous group, ignoring the heterogeneity of anxiety among different types of doctoral students. Third, existing studies have yet to form a comprehensive and universally applicable framework for the influencing mechanisms of anxiety among in-school doctoral students, which is insufficient for deeply guiding the multidimensional causes and intervention strategies for anxiety.

This study aims to clarify the anxiety states of different types of in-school doctoral students and explore the mechanisms underlying their anxiety formation. We intend to address the following research questions: (1) what causes anxiety among different types of in-school doctoral students? (2) How do the factors leading to anxiety in different types of in-school doctoral students exert their effects? (3) How can a framework for the influencing mechanisms of anxiety in different types of doctoral students be established?

Our study will make the following contributions: first, by employing a mixed-methods analysis approach, we will examine the anxiety states of in-school doctoral students. Quantitative analysis will reveal overall trends in anxiety among these students, while qualitative analysis will provide deeper insights into the formation logic and influencing pathways of individual anxiety. This comprehensive perspective, integrating macro-environmental and micro-individual factors, will surpass existing literature by thoroughly analyzing the multiple causes of anxiety among in-school doctoral students. It not only presents the surface characteristics of anxiety among doctoral students in school but also systematically, comprehensively, and accurately explores the underlying deep-seated difficulties behind it. Second, by comparing and analyzing the personalized and differentiated psychological needs of different types of doctoral student groups, we can distinguish the anxiety states and influencing factors of various types of doctoral students in school, go beyond the unified explanation of doctoral student anxiety states in previous studies, and further expand the precise anxiety counseling and psychological support service network for various groups of doctoral students. Third, we will innovate by constructing a framework for the influencing factors of anxiety among in-school doctoral students around factors, such as gender, age, love and marriage pressure, self-awareness, graduation pressure, and employment pressure, thereby forming an intervention mechanism. Unlike existing frameworks for analyzing factors influencing anxiety, this mechanism not only explores the ways in which macro- and micro-level factors affect anxiety but also presents the complexity and diversity of anxiety among different types of doctoral students by analyzing the interaction relationships between these factors. At the same time, based on this mechanism, we aim to investigate the intervention focus of various types of anxiety among doctoral students, in order to enable the effective establishment of dynamic coordination pathways for the prevention, identification, monitoring, and assessment of anxiety within the doctoral student population.

## 2. Literature Review

By conducting a retrieval and synthesis of relevant literature on “anxiety among doctoral candidates in universities”, we aim to delve deeply into and analyze the complex and multidimensional psychological phenomenon. The objective is not only to elucidate the significance of our attention to the issue of anxiety among doctoral candidates in universities but also to integrate existing macro-level and micro-level research findings, thereby revealing the severe consequences and underlying realities of anxiety prevalent within this doctoral population. Furthermore, we endeavor to identify both the commonalities and differences in the manifestation of anxiety among various types of doctoral candidates through a comparative analysis. Initially, we explore the important reasons why the issue of doctoral student’s anxiety is regarded as a key topic for academic research on individual mental health care for researchers and the sustainable development of scientific research in the current global scientific research ecosystem. Secondly, we integrate a dual perspective of the macro academic environment and micro academic practice to examine the multidimensional real roots of anxiety among doctoral students. Thirdly, we should break away from the inherent thinking of focusing on the common needs of doctoral students’ anxiety from a similarity perspective and choose to explore the significant characteristics of anxiety psychology generation among different types of doctoral students based on the differences in their learning styles.

### 2.1. Why Explore the Anxiety of Doctoral Students

**Figure 2:**
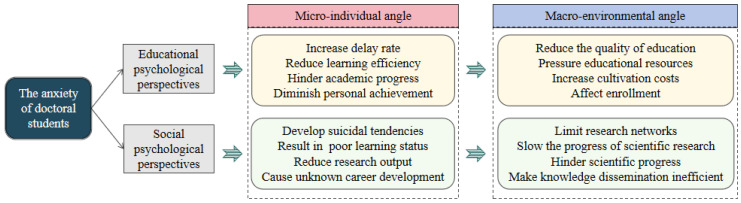
The causes of anxiety for doctoral students.

Based on the promotion of education and the rapid development of society, this study explores the importance of focusing on the anxiety experienced by in-school doctoral students. Utilizing a point-to-surface analytical approach, it comprehensively addresses the serious consequences related to their anxiety from both micro-individual and macro-environmental angles, as illustrated in Figure 2.

First, from the perspective of the negative impact of the anxiety among doctoral students on the advancement of educational endeavors, the anxiety of in-school doctoral students not only hinders their academic progress at the micro-individual level, but collective anxiety, at the macro-educational environment level, contributes to a decline in educational quality and a waste of educational resources, affecting the flourishing development of higher education. At the micro level of educated individuals, doctoral students often face multiple pressures from academic courses, article publication, and dissertations (Gewin, 2012; Park et al., 2021; Reay, 2018). Evans et al. (2018b) found that 82% of doctoral students endure moderate-to-severe stress levels. Due to the significant correlation between anxiety and stress levels among current doctoral students, anxiety under high pressure causes physiological and psychological discomfort, reducing learning efficiency, hindering academic progress, and diminishing personal achievement, directly affecting their academic development and innovation (Park et al., 2021). At the macro level of the educational environment, group-anxiety-induced academic difficulties increase doctoral program delays, as evidenced by Pearson’s (2012) study, which found that graduate students have higher attrition rates than undergraduates. At the doctoral level, Stubb et al. (2011) found that 43% of doctoral students considered discontinuing their studies, and Anttila et al. (2015) reported that 56% of doctoral students had considered dropping out during their dissertation-writing process. Those who considered discontinuing their studies experienced greater stress, anxiety, and fatigue (Anttila et al., 2015; Pyhältö et al., 2012). Maher et al. (2020) directly linked anxiety in doctoral students to their decision to leave their programs. The phenomenon of delay caused by anxiety not only further increases the psychological burden of doctoral students but also puts pressure on the educational resources and management of graduate training institutions (H. Li, 2012). Specifically, this raises operational costs in resource allocation, administrative management, and degree awarding, overburdening faculty time and energy, impacting the quality and efficiency of higher education. High attrition rates under high anxiety levels might be perceived by future students and the public as a failure of universities to meet student needs, affecting enrollment and higher education development (Pauley et al., 1999).

Second, from the perspective of the negative impact of the anxiety among doctoral students on the substantial progress and development of society, as a pivotal force in scientific and technological progress and innovation demanded by society, doctoral candidates on campus experience anxiety that not only results in instability in the output of scientific and technological achievements and uncertainty in career development at the individual level, potentially even nurturing societal issues, such as suicide, but also impedes the efficient promotion and steady development of technological innovation at the societal level by diminishing the efficiency of individual contributions to science and technology. Scientific research is a pivotal form of knowledge wealth driven by societal needs, with knowledge-based economies relying on human and social capital generated by knowledge workers for growth and prosperity, particularly in higher education, for developing new knowledge practices (Davis et al., 2006; Maqsood et al., 2019). Doctoral students are central to the evolving relationship between universities and their environments, facilitating knowledge transfer between academia and industry (Thune, 2009). Their work is essential for scientific progress, with doctoral degrees being prerequisites for knowledge production and transformation in higher education, crucial for societal development in the knowledge economy era (Levecque et al., 2017; F. Li et al., 2022). Consequently, the public holds high expectations for researchers’ social responsibility, viewing doctoral students as an elite group endowed with exceptional talents and skills, leading in social participation and recognition (Chen, 2021). However, at the micro level of educated individuals, firstly, under the high expectations of society, universities have increased assessments of doctoral students’ research outcomes, raising demands for their research capabilities. The dual social pressure of interpersonal relationships and financial conditions has led to obstacles, such as loneliness, anxiety, irritability, and fatigue in the process of completing a doctoral degree (Ahmad et al., 2023; Cornwall et al., 2019; Schmidt & Hansson, 2018). Students who feel extremely anxious are even prone to suicidal thoughts, which in turn triggers a series of thorny social issues related to the individual life, health, and well-being of doctoral students (Garcia-Williams et al., 2014). It also touches upon issues, such as the deterioration of the technology research and development environment, the lack of social awareness of the mental health of highly educated talents, and the sustainability of the higher education system and the overall harmony and stability of society. Secondly, there is an imbalance in the supply and demand of doctoral talents, with a shortage of academic positions in the labor market (Chen, 2021). Fuhrmann et al.’s (2011) survey revealed that doctoral students have broad considerations for career choices but lack confidence in their current options, exacerbating employment anxiety. At the macro level of the social environment, in an unstable competitive environment for academic and career development, the gap between doctoral expectations and reality results in dissatisfaction with research experiences and a poor learning status, increasing research anxiety, hindering the completion of research tasks, and affecting the quality renewal of research networks, innovation in scientific research, and efficient dissemination of scientific knowledge in the process of social development, making it difficult to effectively meet the technological level required for rapid social development (Zeng & Zhang, 2023).

### 2.2. The Difference in Anxiety for Different Types of Doctoral Students

**Figure 3:**
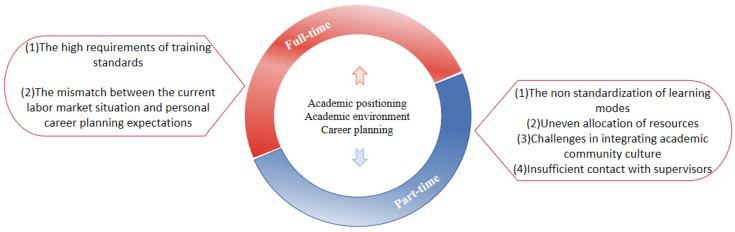
The difference in anxiety for different types of doctoral students in school.

Due to the inherent differences in academic positioning, the academic environment, and career planning (Figure 3), there are significant differences in the anxiety expression and sources between full-time and part-time doctoral students.

#### 2.2.1. The Formation of Anxiety Among Full-Time Doctoral Students

The anxiety experienced by full-time doctoral students mainly stems from the high requirements of training standards, the mismatch between the current labor market situation, and personal career planning expectations. Firstly, in terms of academic positioning, Offerman (2011) noted that full-time doctoral students have clearer academic development goals and can fully immerse themselves in research, adapting to academic culture and networks. Full-time doctoral students primarily bear the responsibility of academic practice and the transmission of research, which enables them to invest more energy in learning and research activities, focusing on becoming high-level academic talents with strong research and innovation capabilities. This group has been given more stringent academic requirements by universities, and academic competition has become a primary stressor for doctoral students (Liu & Gao, 2024). Their anxiety is more related to research pressures and academic demands. Secondly, in terms of employment opportunities, part-time doctoral students often have stable jobs and rich practical experience, with career development being a key factor in their decision to start and continue their doctoral studies, less affected by labor market changes (Mills et al., 2014). Full-time doctoral students usually devote themselves full-time to learning and research, and relatively lack career planning compared to part-time doctoral students (Chen, 2021). Their career development faces greater uncertainty. The academic job market is highly saturated, and they have limited knowledge of non-academic employment opportunities, leading to prolonged transitions from completing their degrees to stable employment, which undoubtedly increases their employment anxiety (Kehm, 2009; Walters et al., 2021). Finally, in terms of employability, full-time doctoral students show more obvious anxiety experiences compared to working doctoral students or doctoral students with work experience. Most full-time doctoral students say that they have never received systematic training in job seeking skills and abilities because they choose to continue their studies after graduating from undergraduate and master’s degrees. The lack of standardized resumes and tense performance during the interview process affect their ability to fully express themselves, which in turn affects their job search results (Feng & Zhang, 2017). Faced with insufficient returns on educational investment and uncertain future development, full-time doctoral students without clear job prospects exhibit higher psychological pressure and anxiety than their part-time counterparts who are familiar with their work positions.

#### 2.2.2. The Formation of Anxiety Among Part-Time Doctoral Students

The main sources of anxiety faced by part-time doctoral students can be attributed to the non-standardization of learning modes, uneven allocation of resources, challenges in integrating academic community culture, and insufficient contact with supervisors. Firstly, in terms of academic positioning, many part-time doctoral students pursue their degrees for career development and promotion, displaying more utilitarian learning motivations, so their anxiety primarily stems from balancing work and study (Cai et al., 2020). Part-time doctoral students often juggle dual roles as researchers and workers, simultaneously managing academic research, work practices, and family responsibilities. Balancing all of these roles often requires sacrifices and compromises (Gardner & Gopaul, 2012). They face significant obstacles in pursuing their degrees due to the “insufficient study time” granted by their employers (Mills et al., 2014). The need to balance personal and professional matters affects their study behavior and development as researchers, leading to anxiety about completing their doctoral degrees amidst time conflicts and energy dispersal (Watts, 2008). As they attempt to balance limited time between family and school, they may experience negative emotions, such as guilt, worry, anxiety, and anger (Lu et al., 2020). Secondly, in terms of resources and the environment, institutions may support full-time students more, anticipating quicker completion of their studies to enhance the institution’s research image (Watts, 2008). Offerman (2011) also found that, compared to full-time doctoral students who enjoy sufficient academic support, part-time doctoral students lack adequate resources for academic development, leading to feelings of alienation and marginalization. This limits their learning opportunities and deep engagement in academic research, diminishing their academic enthusiasm and participation in academic activities, thereby causing anxiety. Furthermore, in terms of the cultural environment, part-time doctoral students face challenges due to full-time jobs and geographic distance, struggling to engage with peer and academic cultures (Deem & Brehony, 2000; Niemczyk, 2016). This cultural disconnection hinders their development, reducing their sense of academic identity and belonging, leading to anxiety about handling rigorous academic tasks alone (Watts, 2008). Finally, in terms of the guidance environment, full-time doctoral students maintain higher communication frequency with their advisors, while part-time doctoral students have less face-to-face interaction, making it challenging to develop productive and engaging supervisory relationships (Watts, 2008). Balancing work or family obligations, they find it difficult to connect with advisors, facing power asymmetries with their employment leaders, resulting in excessive work and study stress (Mills et al., 2014; Offerman, 2011). Under dual pressures, part-time doctoral students struggle with insufficient advisor guidance, failing to respond effectively to academic tasks, thus experiencing confusion and anxiety amid unstable academic progress.

### 2.3. Influencing Factors of Anxiety of Doctoral Students

**Figure 4:**
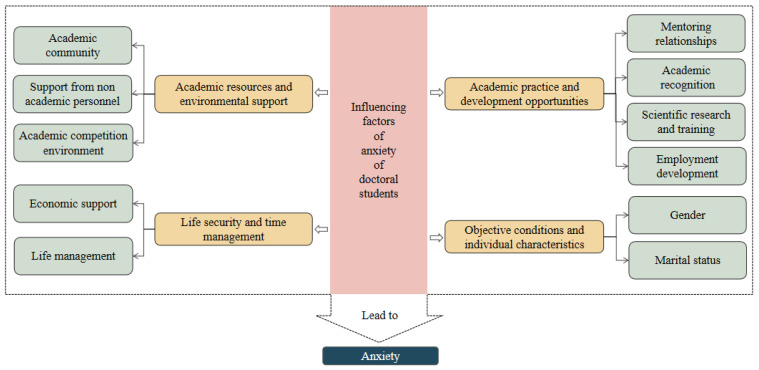
Analysis of influencing factors of anxiety for doctoral students.

The emergence of anxiety among doctoral students, as illustrated in Figure 4, stems from a combination of macro-level constraints, such as limited academic resources and environmental support, and micro-level challenges, including difficulties in academic practice, uncertainties surrounding future development opportunities, inadequate living safeguards, and personal deficiencies in various aspects. In the formation process of doctoral students’ anxiety, academic resources and environmental support are key constraints, academic practice and development opportunities are the core roots, life security and time management are important incentives, and objective conditions and individual characteristics are potential foundations. These factors interact with each other and collectively stimulate the emergence of anxiety among doctoral students in school.

#### 2.3.1. Academic Resources and Environmental Support

Obtaining academic resources and adapting to the environment are one of the basic needs for doctoral students’ learning and life. When doctoral students find themselves in a state of resource scarcity and an unfavorable academic environment, they may experience feelings of helplessness and confusion, which can lead to anxiety.

Firstly, starting from the academic community, it is crucial for the mental health and well-being of doctoral students in their early stages (Jackman et al., 2022). Stubb et al. (2011) and Schmidt and Hansson (2018) both emphasize the dual function of the academic community for doctoral student groups; the latter divides the doctoral student group into two categories: those who empower the academic community (similar to the experience of the academic community, that is, supporting their own learning and growth as a researcher) and those who see the academic community as a burden (negative description of their relationship with the academic community, or a lack of relationships). Doctoral students who empower the academic community will experience less anxiety during their doctoral studies, as the sense of empowerment is negatively correlated with anxiety, while the negative attributes of dissatisfaction with the academic research atmosphere are positively correlated with anxiety (Anttila et al., 2015). Secondly, from the perspective of support from non-academic personnel, this is an important support for doctoral students to carry out academic research smoothly. Waheed et al. (2021) emphasized the administrative support of department heads, college leaders, and assessment officers, as well as the lack of proactive follow-up mechanisms from external evaluations and administrative departments, which have an important impact on the delay in submitting doctoral theses and waiting for thesis evaluations. The delay in submitting papers and the uncertainty of the evaluation process make them anxious. Thirdly, at the level of the academic competition environment, the competitive atmosphere can create a high level of pressure for doctoral students (Kurtz-Costes et al., 2006). Academic demands for competition ferment in a mixed emotion of loneliness, high expectations, and insomnia, these emotions can evolve into debilitating depression, painful anxiety, and even suicide attempts (Gewin, 2012).

#### 2.3.2. Academic Practice and Development Opportunities

The level of academic practice and employment-development opportunities constitute crucial pillars for the academic growth of doctoral students. Inadequate high-intensity research training can lead to a sense of frustration among doctoral candidates, while the limited applicability of academic skills in the job market may create a perception of bleak prospects, thereby exacerbating their anxiety.

Firstly, from the perspective of mentoring relationships, interpersonal relationships can sometimes serve as a support and coping mechanism, but they can also be a source of stress at times (Cornwall et al., 2019; Schmidt & Hansson, 2018). In the relationship between advisors and advisees, the advisor is the main provider of support for doctoral students in the academic community, but at the same time, potential conflicts between doctoral students and advisors can cause doctoral students to experience anxiety (Jackman et al., 2022; F. Li et al., 2022). Liu et al. (2019) found significant differences in the frequency of meetings between research students with different levels of anxiety, and the advisor–advisee relationship can play a partial mediating role in the relationship between doctoral students’ self-efficacy and anxiety impact. Singh’s (2020) research results show that doctoral students who perceive themselves to have a strong relationship with their mentors have lower scores on anxiety scales than those who perceive themselves as having a weak relationship with their mentors. Starting from the relationship between mentors, Waheed et al. (2021) found that professional jealousy can lead to obstacles and delays in the submission of doctoral theses and after submission, which relates to the anxiety of doctoral students. In addition, doctoral students are under pressure to have an impact, attract the public’s attention, shine on social media, and influence policies (Reay, 2018). If doctoral students face a gap between their own expectations and the reality of their mentors’ expectations, they may feel anxious while trying to meet their mentors’ expectations (S. Zhang et al., 2022). Secondly, from the perspective of academic recognition, Liu et al.’s (2019) research shows that there is a significant negative correlation between doctoral students’ self-efficacy and anxiety; the role of motivation has been emphasized in several studies on the relationship between doctoral study and doctoral student anxiety, and a lack of interest is positively correlated with anxiety (Anttila et al., 2015; Marais et al., 2018; Stubb et al., 2011; Sverdlik et al., 2018). Thirdly, from the perspective of scientific research and training, frequent assessments, low status and high workload, and deadlines are all sources of pressure for doctoral students; the uncertainty of research can, to some extent, trigger anxiety in doctoral students (Kurtz-Costes et al., 2006; F. Li et al., 2022; Schmidt & Hansson, 2018). Liu et al.’s (2019) research indicates that graduate students with different levels of anxiety have significant differences in the difficulty of publishing papers. The research results of Park et al. (2021) on a graduate student population with 77.4% of doctoral students also showed that the graduation thesis, thesis, and other research have become the primary pressures for graduate students, and stress and anxiety show a significant positive correlation. Sverdlik et al. (2020) found that subjective academic belonging is a negative predictor of imposter syndrome, which predicts higher levels of depression, stress, and illness symptoms. In summary, the above studies point out that doctoral students need to meet high standards of publication and scientific research in order to meet graduation and employment criteria. Such high standards tend to cause self-doubt among doctoral students, which makes imposter syndrome widely appear in doctoral students, who believe that their academic achievements are due to chance or external factors, rather than personal strength, and then worry that they are insufficient to support their academic status. Fourthly, from the perspective of employment development, fear of failure and low expectations of long-term career development and job security can easily lead to anxiety (F. Li et al., 2022). Marais et al. (2018) found that, compared to the effect of mentors on doctoral happiness, career training and prospects have a greater impact on the psychological health of doctoral students.

#### 2.3.3. Life Security and Time Management

The adequacy of life support is an important prerequisite for doctoral students to efficiently complete their learning and research tasks. The scarcity of economic resources and the conflicts in scheduling among academic, work, and personal life can lead them to a dilemma of lacking material and spiritual support.

Firstly, from the perspective of economic support, numerous studies have pointed out the significant impact of economic issues on the development and psychological status of doctoral students (Cornwall et al., 2019; Kurtz-Costes et al., 2006; Sverdlik et al., 2018; Schmidt & Hansson, 2018; Szkody et al., 2023). Acker and Haque (2015) believe that funding can affect the extent to which doctoral students integrate into the departmental community, which in turn affects their “social” level. F. Li et al. (2022) found that the fit between funding support and doctoral students’ psychological health was significantly positively correlated, while Park et al.’s (2021) research results indicate that there is no significant difference in economic pressure between master’s and doctoral students. Secondly, from the perspective of life management, time issues and family issues are important sources of pressure for doctoral students (Schmidt & Hansson, 2018). Several scholars mentioned the balance and conflicts among work, family, and doctoral studies in their research (Cornwall et al., 2019; Levecque et al., 2017; F. Li et al., 2022). Liu et al.’s (2019) research indicates that research students with different levels of anxiety have significant differences in balancing work, family, and doctoral project difficulties.

#### 2.3.4. Objective Conditions and Individual Characteristics

Individual variations in characteristics can lead to different reactions of doctoral students to the same event, resulting in different psychological perceptions and emotional experiences, which is an inherent element in the formation of their anxiety.

Firstly, from a gender perspective, different scholars have found in their research that the anxiety levels of female doctoral students are higher than those of male doctoral students (F. Li et al., 2022; Sverdlik et al., 2023). Evans et al. (2018a) also found similar results in their research on the graduate population (with doctoral students accounting for 90%). However, based on an analysis of research data from Finland, Stubb et al. (2011) found no significant differences between male and female doctoral students in anxiety about their own experiences but instead found that male self-assessed fatigue was significantly higher than female self-assessed fatigue. Secondly, from the perspective of the marital status, Hyun et al. (2006) found that graduate self-assessed psychological health needs were significantly negatively correlated with their economic confidence in completing graduate studies and their married status. But Park et al.’s (2021) research results showed no significant difference in economic pressure between single and married groups in the graduate population (77.4% were doctoral students). Singh’s (2020) study also indicated no significant correlation between doctoral students’ marital status and anxiety. In addition, the physical health status is also a key factor in the anxiety of doctoral students (F. Li et al., 2022; Marais et al., 2018).

## 3. Methodology

This study was approved by the Ethics Review Committee of the School of Education of Beijing Institute of Technology and strictly adheres to ethical principles. All subjects who participated in the study gave informed consent and voluntarily participated in the study.

### 3.1. Data

The data in this study come from two main sources: survey questionnaires and semi-structured psychological interviews.

The specific conditions of the research subjects in the questionnaire are shown in Table 2. This study used a stratified random sampling method to distribute survey questionnaires to 700 doctoral students from three universities in Beijing, China, through an online platform, to distinguished the differences in doctoral students’ anxiety from four dimensions, such as gender, age, study mode, and grade. A total of 638 valid questionnaires were collected, with an effective rate of 91.1%. Among the valid sample, at the gender level, there were 402 male doctoral students and 236 female doctoral students; at the age level, there were 102 doctoral students aged 25 years old or younger, 397 doctoral students aged 26–29, 81 doctoral students aged 30–34, 50 doctoral students aged 35–39, and 8 doctoral students aged 40 or older; in terms of study methods, there were 552 full-time doctoral students and 86 part-time doctoral students; at the grade level, there were 143 first-year doctoral students, 121 second-year doctoral students, 141 third-year doctoral students, 145 fourth-year doctoral students, 59 fifth-year doctoral students, and 29 sixth-year or higher doctoral students. We investigated the anxiety status of doctoral students based on the generalized anxiety disorder-7 item (GAD-7), which is a self-report scale used to measure the severity of anxiety. Its scientific validity has been verified in adults and Chinese teenagers (Sun et al., 2021). It has good reliability and validity and has been widely used in anxiety-related research at home and abroad. It has multiple advantages, such as professionalism, universality, applicability, and simplicity, and it is conducive to comprehensively and accurately reflecting the anxiety status of doctoral students. The scale includes seven items and adopts a 4-level score; 0–3 points are given from “completely unable” to “almost every day”. The higher the score, the higher the anxiety level. According to different score levels, the anxiety level is as follows: ≤4 points, indicating no anxiety; 5–9 points, indicating mild anxiety; 10–14 points, indicating moderate anxiety; 15–21 points, indicating severe anxiety, and a total score of ≥10 points is defined as clinical positive anxiety (He et al., 2010).

The specific conditions of the research object of the psychological interview are shown in Table 3. This study, based on the questionnaire survey, selected 10 doctoral students of different gender, age, grade and major categories from universities in Beijing, China, for in-depth interviews to understand the current development, stress, and expectations of doctoral students and to explore the relevant factors influencing their anxiety. To ensure the rationality and scientificity of the research, the participants involved doctoral students of different genders, ranging in age from 25 to 33, covering various disciplines, such as natural sciences, engineering, humanities, and social sciences. During the interviews, the participants underwent 30–60 min of semi-structured questioning. We recorded the participants’ feelings and suggestions using recording devices, strictly adhered to the participants’ informed consent, promised to strictly keep the interview content confidential, and respected the rights of the participants.

### 3.2. Method

This study employs a mixed research method design, combining and transforming quantitative and qualitative analyses to comprehensively understand the relationship between anxiety levels among doctoral students and the related factors, with a focus on the underlying mechanisms behind anxiety (Lee Cunningham et al., 2023). According to Creswell and Creswell’s (2005) research, the integration of generalizable trends captured by quantitative data with the more in-depth insights offered by qualitative data within specific contexts can provide a more comprehensive set of information than what either quantitative or qualitative data alone would be able to offer. The triangulation mixed methods design collects quantitative and qualitative data at the same time, so that researchers can convert the data, so as to compare the detailed situational qualitative data with more standardized quantitative data. Consequently, this method integrates the strengths of both quantitative and qualitative research, enabling it not only to utilize a wide range of data to reveal the general characteristics of doctoral students’ anxiety phenomena, to delineate distribution patterns across diverse doctoral cohorts, and to discern potential trends in its manifestation, but also to overcome the limitations of data types and explanatory power that understand the understanding of the interviewed doctoral students on anxiety, so as to summarize the doctoral students’ personal experience and cognitive understanding of anxiety, explore the deep-seated causes of their anxiety, and draw more reliable and scientific research conclusions (Koerner, 2014).

Firstly, we use a statistical analysis to present the basic situation of anxiety among doctoral students in the survey questionnaire, observe the perception of anxiety levels among doctoral students under different conditions through differential analysis, reveal the overall characteristics influencing doctoral students’ anxiety, and explore the evolutionary trends of their anxiety states. However, quantitative research examines the factors influencing doctoral students’ anxiety based on their basic conditions, without comprehensively considering the stimulating effects of their internal psychological conditions and external environmental conditions. It also fails to deeply explain the diverse influencing factors and mechanisms behind doctoral students’ anxiety from the perspective of individual doctoral students. To address these shortcomings, we follow the approach of “understanding the current status of anxiety—clarifying the influences of anxiety—investigating the causes of anxiety”, and further use in-depth interviews to flexibly obtain a large amount of intuitive information on the anxiety of doctoral students. We delve into their internal motivations and emotions and search for the deep-seated real and psychological factors behind their anxiety. We analyze the interview texts deeply using grounded theory, comprehensively organize, analyze, and summarize the data using NVivo14.0 software, use three-level coding to form abstract concepts from the interview content, systematically refine the core influencing factors of anxiety among doctoral students, and combine the interview texts to outline the influencing factor conceptual framework, as follows:

#### 3.2.1. Open Coding

First, the basic information of the 10 interviewees and the author’s interview notes were organized. Based on this, the interview recordings were transcribed verbatim, and irrelevant content in the interviews was removed, resulting in a transcription text of nearly 40,000 words; 193 representative descriptive texts were extracted, and 34 initial concepts and 14 conceptual categories were identified, as illustrated in Appendix A.

#### 3.2.2. Axial Coding

Building upon the open coding, axial coding was conducted to analyze the relationships between various conceptual categories. Similar concepts and threads were further summarized and merged to assign higher-level conceptual names that are more comprehensive. At this stage, four main categories were formed, further identifying the factors influencing the anxiety of doctoral students, as illustrated in Appendix B.

#### 3.2.3. Key Code

**Figure 5:**
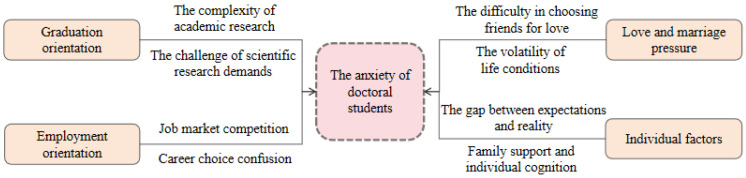
Conceptual framework of factors influencing the anxiety of doctoral students in school.

In this study, the “anxiety of doctoral students in school” as the direct research object, emphasizes the anxiety emotion and psychological state of doctoral students in school in the aspects of study, life, employment and so on. We regard it as the core category, aiming to deeply explore the multidimensional causes and influencing paths behind this complex psychological state and provide an in-depth analysis for understanding the overall anxiety state of different types of doctoral students in school. Based on this, this study deeply analyzes the four main genera obtained from the spindle coding. Graduation orientation, employment orientation, love and marriage pressure, and individual factors are direct influencing factors of anxiety in doctoral students, each associated with several other conditions, collectively affecting the psychological state of doctoral students. This forms the conceptual framework of factors influencing the anxiety of doctoral students, as shown in Figure 5. The specific influencing paths are as follows: the complexity, urgency, and challenges of academic research under the graduation orientation lead to anxiety in doctoral students; market competition, choice confusion, and economic burden under the employment orientation exacerbate anxiety in doctoral students; the instability of life status behind marriage and relationship pressure is an emotional factor in the anxiety of doctoral students; the external expectations, reality gaps, and self-state involved in individual factors are the internal roots affecting the anxiety of doctoral students.

#### 3.2.4. Coding Reliability and Theoretical Saturation Test

After the coding process, contact was made again with the interviewees. All 10 interviewees agreed to evaluate the coverage of coding regarding their experiences and circumstances, indicating that the coding results accurately reflected their real situations and feelings. This judgment indicates good coding reliability. Furthermore, the theoretical saturation of this study was tested by analyzing the interview records of an additional three doctoral students. The results showed that no new categories emerged regarding the factors influencing doctoral students’ anxiety, except for graduation orientation, employment orientation, marriage and relationship pressure, and individual factors. Therefore, it was determined that theoretical saturation had been achieved.

## 4. Result and Discussion

### 4.1. Quantitative Result

Our quantitative results will be presented in two parts: the overall status of anxiety among doctoral students in school and the differences in anxiety among doctoral students with different demographic characteristics. These results help us reveal the overall trend in anxiety among doctoral students, proving that anxiety symptoms are prevalent in this group and highlighting the differences in anxiety among doctoral students with different demographic characteristics. This insight guides us to conduct interviews for a deeper analysis of the current status and causes of anxiety among different types of doctoral students.

**Figure 6:**
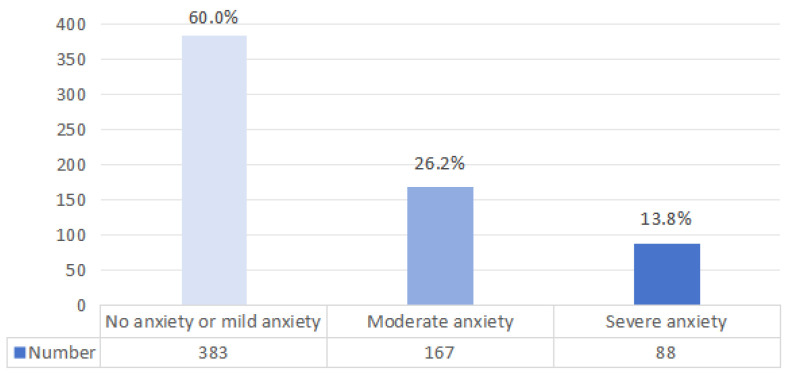
Anxiety level of doctoral students.

As shown in Figure 6, 40% of doctoral students exhibit a relatively high level of anxiety (total score ≥ 10), with 167 students experiencing moderate anxiety (total score of 10–14), accounting for 26.2%, and 88 students experiencing severe anxiety (total score of 15–21), accounting for 13.8%. The highest anxiety score among doctoral students was 21, the lowest was 0, and the average score was 11.037. Regarding the overall status of anxiety among doctoral students, the phenomenon of anxiety is relatively common, consistent with the conclusions of Marais et al. (2018), which found that the most important factors contributing to the immense pressure on doctoral students are closely related to school and education. The uniqueness of doctoral education results in significant pressure on dissertation publication and employment, compounded by economic pressure and the uncertainty of returns on educational investment, leading to widespread anxiety among doctoral students in school.

The analysis results of differences in anxiety among doctoral students with different demographic characteristics are shown in Table 4. Significant differences in anxiety are observed concerning gender, age, grade, marital status, and group meeting frequency. The specific analysis results are as follows:

(1) Gender. Male doctoral students (N = 402) have an average anxiety score of 11.056, while female doctoral students (N = 236) have an average score of 11.614, indicating a significant difference in anxiety levels, with female doctoral students exhibiting significantly higher anxiety levels than their male counterparts. This suggests that female doctoral students are more susceptible to anxiety in both academic and personal life. The group of female doctoral students confront unique anxieties related to mate selection, childbirth, and employment. In the context of marital relationships, societal traditions impose constraints on women, with those who remain unmarried by the age of 30 being labeled as “leftover women”, thereby experiencing heightened pressure in the realm of romance. The best childbearing age for women is also before the age of 30, and the vast majority of female doctoral students will reach the age of 30 after finishing their studies. The uncertainty of marriage and childbirth will put them under greater pressure. From an employment perspective, women also encounter unfair treatment and greater obstacles due to age limitations or gender-based disadvantages. In terms of role perception, as per the research conducted by Carter et al. (2013), the transformation of identity that female doctoral students undergo during their pursuit of a Ph.D. seems to clash with societal expectations of their roles outside the academic setting. To fulfill their academic commitments, these women may need to renegotiate their roles, with the “good” woman selflessly prioritizing the needs of her family over her own aspirations, while the “excellent” doctoral student meets deadlines, remains focused on her research, and achieves her goals. Under the pressure of role balance and choice, female doctoral students often fall into the anxiety cycle of achieving self-identity, realizing personal value, and undertaking family responsibilities.
(2) Age. Doctoral students under 30 years old (N = 499) have an average anxiety score of 11.060, whereas those aged 30 and above (N = 139) have an average score of 11.563. There exists a significant difference in anxiety levels across age groups, with older doctoral students experiencing higher anxiety levels. First, the time cost is an important investment for doctoral students. The older the doctoral students are, the greater the employment, family, and economic pressure they face. The traditional concept of “establishing oneself at thirty” contributes to increased pressure for stable employment and life status, intensifying anxiety among older doctoral students. Second, Y. Zhang (2024) found that the younger the entry age of doctoral students, the more likely they are to win national scholarships and graduate successfully. Compared with young doctoral students, older doctoral students may worry that they cannot adapt to the high-intensity and rigorous scientific research life as quickly as young students and then worry that their academic output cannot match that of young students, facing the potential risk of a decline in academic competitiveness. Therefore, older doctoral students pursue academic achievements and doctoral degrees more intensely, but they can pay less energy than younger students. This mismatch also leads to dissatisfaction with their needs and serious anxiety.
(3) Grade. First- and second-year doctoral students (N = 264) have an average anxiety score of 10.923, while third-year and above doctoral students (N = 374) have an average score of 11.569. Significant differences exist in anxiety levels across different grades, with higher-grade students experiencing significantly higher anxiety. Currently, the global doctoral education generally presents the trend of high-quality training and high standard graduation. Senior doctoral students are facing multiple pressures, such as academic achievements, graduation theses, employment, and so on. Some students have even delayed graduation, which leads to self-doubt and anxiety. As the research of Zhao et al. (2020) pointed out, due to the graduation and employment of some senior doctoral students, their academic attitude is good, but they lack enough time for academic paper publication and are prone to academic confusion, reduced self-efficacy, and other difficulties.
(4) Marital Status. Different from Feng (2021), unmarried doctoral students in this study (N = 507) have an average anxiety score of 11.467, while married doctoral students (N = 131) have an average score of 11.024. The calculated Cohen’s d value is 0.482, indicating that the difference between the two is medium to small. Since Hyun et al. (2006) has found that there is a significant negative correlation between the mental health needs of graduate students and the married status and the interview also pointed out that love and marriage pressure is an important source of anxiety for unmarried doctoral students, we believe that doctoral students who are married or not have a significant difference in anxiety level. The anxiety levels of unmarried doctoral students were significantly higher than those of married doctoral students. Stable intimate relationships provide emotional and financial support, which aids in alleviating anxiety among married doctoral students. Focusing on the perspective of having or not having children; doctoral students with children (N = 56) have an average anxiety score of 11.572, while those without children (N = 582) have an average score of 11.001. No significant difference in anxiety levels is observed based on the parenthood status.
(5) Group Meeting Frequency. Doctoral students who attend group meetings at least once every two weeks (N = 309) have an average anxiety score of 10.349, while those with less frequent meetings (N = 329) have an average score of 11.781. Significant differences exist in anxiety levels based on the group meeting frequency, with more frequent meetings correlating with lower anxiety levels. This indicates that regular group meetings effectively alleviate anxiety by facilitating communication with advisors, clarifying research directions, and resolving academic challenges. As C. Li (2022) highlighted in his study, regular laboratory group meetings are a “safe place” to test doctoral students’ academic ideas and views, which helps doctoral students improve their ability to analyze and solve problems in the process of being questioned and criticized.

**Table 4:** Comparison of variability based on different demographic profile variables of anxiety among doctoral students.

Character		Levene’s Variance		Number	Average Score (SD)	Sig.	T-Value	Cohen’s d
		F	*p*					
Gender	Male	0.289	0.594	402	11.056 (0.936)	0.000	−5.386 ***	−0.554
	Female			236	11.614 (1.073)			
Age	<30	0.291	0.345	499	11.060 (0.923)	0.000	−4.981 ***	1.236
	≥30			139	11.563 (0.896)			
Grade	Grade 1 and 2	0.137	0.678	264	10.923 (0.922)	0.007	−2.182 **	−0.727
	Third-year and above			374	11.569 (0.929)			
love and marriage	Unmarried	0.187	0.701	507	11.467 (0.958)	0.000	3.569 ***	0.482
	married			131	11.024 (0.878)			
Any children	Yes	0.208	0.599	56	11.572 (0.991)	0.131	5.445	\
	No			582	11.001 (0.924)			
Group frequency	Fortnightly and above	0.122	0.496	309	10.934 (0.957)	0.001	−3.140 ***	−0.855
	Less than once a fortnight			329	11.781 (1.023)			

Note: ** *p* < 0.01; *** *p* < 0.005.

### 4.2. Qualitative Result

Based on the axial coding results of psychological interviews, we identified four major categories—graduation orientation, employment orientation, pressure from romantic relationships, and individual factors—that collectively contribute to the formation of anxiety in doctoral students.

#### 4.2.1. Graduation Orientation

Graduation orientation is the primary factor contributing to the anxiety of in-school doctoral students and represents a fundamental need for them. It is primarily manifested in three aspects: advisor tasks and advisor–student relationships, research pressure, and graduation procedures. First, regarding advisor tasks and advisor–student relationships, three doctoral students mentioned that the tasks assigned by their advisors exceeded their capacity to complete them. Additionally, some advisors use the completion of research tasks as a prerequisite for students to start their dissertation and graduate, leading to feelings of passivity and anxiety. As Bireda (2015) found, all participants believed that their tutors were one of the most important factors affecting their doctoral experience. Almost all respondents in this study highlighted that advisors, as the primary person responsible for graduate training, play a crucial role in reducing academic anxiety through good communication, guidance, academic support, and emotional care. In contrast, doctoral students with strained advisor relationships experience more pronounced anxiety and helplessness. For example, doctoral student S1 stated the following in the interview:

“I am very afraid to meet my advisor because no matter how hard I try, he always points out a lot of problems with my work, so I don’t dare to discuss issues with him when I encounter problems in my research. Often, I feel quite helpless.”

Second, regarding research pressure and graduation procedures, almost all interviewees mentioned the significance of publishing both minor and major papers, which is a core source of academic anxiety for doctoral students. There is a general consensus among universities in China regarding the regulations on the doctoral program duration and the maximum period of study, typically spanning 3 to 4 years for the standard program and extending up to 6 years as the maximum limit (T. Wang et al., 2024). This implies that doctoral students need to complete a series of tasks, such as high-quality course learning, experimental research, and thesis writing in a relatively tight time, which is a double challenge to their time management ability and psychological endurance. Publishing several minor papers is a necessary but insufficient condition for applying for a doctoral dissertation defense. Minor papers are evaluated based on quantity and journal ranking. According to the respondents, the minimum graduation requirement is the publication of research in a core journal listed by Peking University, with some schools even requiring publications in SCI or SSCI journals, typically demanding at least two papers. These high and stringent requirements significantly increase academic pressure, leading to publication anxiety. There is a widely acknowledged consensus within the academic community that obtaining a doctoral degree is inherently challenging, and the completion of small research papers, serving as a criterion for doctoral students to successfully progress through their academic pursuits, exerts a direct and significant influence on their levels of anxiety. Moreover, Xie and Xu (2024) found that research pressure and the writing duration of dissertations have a significant positive predictive effect on delayed graduation. Universities require doctoral dissertations to be at least 100,000 words long, and the dissertations must undergo anonymous review by the Ministry of Education. The results of these reviews directly affect the students’ graduation prospects. If the review results are unsatisfactory, doctoral students may need to revise their dissertations for a year before defending again, further contributing to graduation anxiety. Consequently, both the difficulty of publishing minor papers and the stringent requirements for doctoral dissertations are significant sources of anxiety for in-school doctoral students.

#### 4.2.2. Employment Orientation

Factors influencing the anxiety of doctoral students in terms of employment orientation primarily include employment choices, salary expectations, job-seeking disadvantages, and competitive pressure, with significant gender differences observed. First, regarding employment choices, doctoral students face pressures from personal academic pursuits, peer competition, societal expectations, and market acceptance. Under these compounded pressures, many interviewed doctoral students expressed concerns about their academic achievements being insufficient to support their employment development, leading to intense anxiety during the job-selection process. According to the nationwide survey of doctoral graduates conducted by the China Doctoral Education Research Center in 2021, it was revealed that 79.8% of doctoral graduates had secured employment, with 52.6% and 9.1% securing positions in universities and research institutions, respectively. Excluding medical samples, 60.4% and 10.8% of graduates are employed in Universities and scientific research institutions, respectively; that is, a total of 71.2% are employed in academic departments. In universities engaged in the construction of world-class universities, excluding the humanities, the employment rate of doctoral graduates in academic sectors across other disciplines (humanities, science, engineering, medicine, and agriculture) decreased by 0.8 to 8.4 percentage points in 2021 compared to 2017, and the thresholds for elite academic positions continue to rise (Xu et al., 2022). The interview results show that most respondents aspire to work in “universities or research institutions” and hope to settle in top-tier cities, like Beijing and Shanghai. However, they commonly report that the types of jobs available to them are shrinking upon obtaining a doctoral degree. If they are not interested in research work, the advantage of having a doctoral degree is diminished. This uncertainty and challenge in employment choices are significant reasons for the anxiety of doctoral students. Second, regarding salary expectations, according to the research conducted by Xu et al. (2022), the data from the 2021 survey of doctoral graduates in China indicates that, with the exception of doctoral students in the field of social sciences, the proportion of students who prioritize “salary” as a primary consideration in their career choices has shown an upward trend among doctoral graduates across other disciplines. In fact, The study by Luo and Zhang (2023) presents data revealing that the distribution of salary ranges among doctors who graduated within the past decade (2010–2020) is as follows: 0.47% earning between 0–50,000 yuan, 30.82% earning between 60,000–100,000 yuan, 40.45% earning between 110,000–150,000 yuan, 18.25% earning between 160,000–200,000 yuan, and 10.02% earning above 210,000 yuan. The continuous high-intensity academic investment during the doctoral pursuit leads this group to have high expectations for their salary returns. However, when actual salary levels do not meet their expectations, it causes a crisis in self-worth recognition, resulting in anxiety. Almost all respondents reported that their salaries are disproportionate to their efforts. This issue is particularly acute among doctoral students in humanities, who face limited job market demand and unclear career planning, resulting in heightened salary anxiety and pessimism about the return on their educational investment. For example, doctoral student S2 mentioned the following in the interview:

“My senior sister just got employed this year, and her annual salary is only 200,000 RMB, which is what we could earn with a master’s degree. The four years spent pursuing a doctorate don’t seem to provide a salary increase that compensates for those years, and we even lose our age advantage. Perhaps our field is less lucrative, but an annual salary of 200,000 RMB is not enough to survive in Beijing.”

Third, regarding job-seeking disadvantages, biases and stereotypes related to gender significantly limit the career opportunities for doctoral students, particularly for female doctoral students. The inherent disadvantages women face in the workplace are especially pronounced for older female doctoral graduates, who are at the last golden age for marriage and childbirth. Societal expectations for women to balance family responsibilities put them at a disadvantage in the job market, diminishing their competitiveness and job success rate. Gender bias in the labor market results in severe job-seeking anxiety among female doctoral students. Fourth, regarding competitive pressure, the difficulty of translating high-level academic achievements into employment advantages in a competitive job market has become a major source of employment anxiety for the interviewed doctoral students. The current employment landscape is increasing, with more job seekers in top-tier cities having overseas doctoral backgrounds, which undoubtedly increases stress levels and affects their anxiety state. Additionally, some respondents indicated that they have never had work experience or systematic job-seeking training, making it difficult for them to compete in job-seeking skills and abilities. This competitive disadvantage significantly exacerbates the anxiety levels of doctoral students.

#### 4.2.3. Love and Marriage Pressure

Love and marriage pressure is a significant source of anxiety for unmarried doctoral students, manifesting in three aspects: romantic experiences, societal expectations, and mate selection criteria. Currently, the pressure of marriage and love constitutes a significant source of non-academic stress for doctoral students (X. Wang et al., 2016). First, regarding romantic experiences, many single doctoral students interviewed expressed that heavy research pressures leave them with no time for relationships and little desire to interact with the opposite sex. This scarcity of time and energy makes it difficult for them to meet the expectations of finding a partner, leading to emotional instability and uncertainty in future planning. The conflict between academic demands and personal life is a pivotal factor in their anxiety. For instance, doctoral student S3 mentioned the following in the interview:

“I spend all my time in the lab from morning till night, with tasks from both my advisors that I can’t complete. How can I have time for a relationship? It’s too much to ask for! Moreover, our department is like a monastery; I don’t even remember the last time I saw a woman. I worry this might cause problems for me.”

Second, in terms of societal expectations, the conflict between common societal values and the role of doctoral students, particularly among female doctoral students, contributes to romantic anxiety. Unmarried female doctoral students frequently mentioned the pressures from societal expectations, such as the challenge of finding a spouse and the stigma of being labeled as “leftover women.” Female doctoral students, focused on academic research, may find it difficult to marry or even consider childbirth within the socially expected timeframe. These societal pressures increase the confusion and anxiety among female doctoral students. Third, regarding mate selection criteria, doctoral students typically have high expectations for their future partners, emphasizing shared values and compatible capabilities. The difficulty in meeting these idealized expectations can increase feelings of frustration and anxiety in the process of finding a compatible partner. Male doctoral students frequently cited factors, such as “gentle personality”, “younger age”, and “physical appearance”, with educational requirements typically being “at least a bachelor’s degree”. They also favored traditional family roles, where the male focuses on external work and the female manages the household and childcare. Conversely, female doctoral students emphasized “compatible personality”, “potential for growth”, and “genuine care”, with a preference for partners who also hold a doctoral degree. They aspired towards successful careers and often expressed a reluctance to be confined to household roles, with several interviewees indicating they “do not want children.” In a relatively closed social environment, stringent mate selection standards are not only emotional expectations but also psychological burdens, making doctoral students cautious and anxious during the partner selection process. In summary, the mismatch in romantic expectations between male and female doctoral students contributes to anxiety related to romantic relationships.

#### 4.2.4. Individual Factors

Individual factors contributing to the anxiety of doctoral students encompass others’ expectations, peer pressure, family circumstances, and self-perception. First, regarding others’ expectations, doctoral students, as highly educated individuals, face multiple expectations from parents, advisors, and society. Interviewed students indicated that these often unrealistic expectations amplify their anxiety levels. Parents expect their children to secure prestigious jobs and substantial incomes post-graduation; advisors expect students to possess strong academic skills and execution capabilities; under the market preferences within specific socio-economic contexts, there is a growing demand for high-skilled talent in non-academic sectors, resulting in the phenomenon where more than half of doctoral graduates worldwide seek employment in the non-academic market, highlighting the existence of a dual labor market for Ph.D. holders (Gu et al., 2018; Zhu et al., 2024). Society expects high-degree holders to contribute significantly to national development. The disparity between these expectations and the students’ current situations often leads to increased anxiety. Second, in terms of peer pressure, doctoral students, situated in elite learning environments, frequently worry about their academic abilities, career prospects, and social value. This anxiety is exacerbated by the highly competitive learning mode. Additionally, some students compare themselves to peers who did not pursue a doctorate. When faced with career and social status comparisons, doctoral students can experience self-doubt and anxiety. For example, doctoral student S4 mentioned the following in the interview:

“I often question whether I made the right choice. My high school classmate, who didn’t perform as well academically, graduated with a master’s degree and started working at a top company. In just a few years, he has enough for a down payment on a house in Beijing, is married, and has children. He’s got it all. Meanwhile, I don’t even know if I’ll ever achieve that kind of life, and if I do, I’ll be almost 40 by then.”

Third, regarding family circumstances, financial support is crucial for doctoral students to continue their research and cover living expenses. Some students interviewed mentioned significant financial pressures within their families, which exacerbates their distress. The lack of effective financial support amplifies feelings of helplessness, affecting academic performance and adding psychological stress and anxiety. The majority of doctoral students’ income comes from stipends, which are limited and barely cover basic on-campus expenses. Given their busy academic schedules, students have little time or opportunity to earn additional income, adding to their stress and anxiety. Fourth, in terms of self-perception, the doctoral journey is an exploration and enhancement of the self. Students have high expectations for their academic achievements. However, the hurdles and reversals encountered in academic research can affect their progress, leading to confusion and anxiety. Nearly all interviewees reported experiencing the discrepancy between “elite ideals” and reality during their doctoral studies. The process of pursuing a degree involves self-doubt, internal conflict, and self-denial. The mismatch between their expectations and reality contributes significantly to their anxiety.

## 5. Conclusions

**Figure 7:**
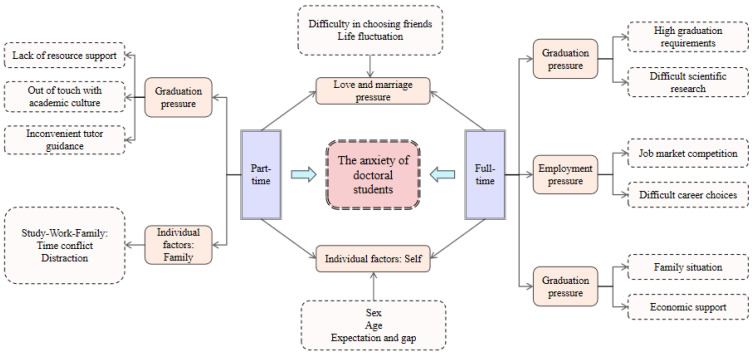
Intervention mechanism framework of anxiety of doctoral students.

This study focuses on the anxiety levels of doctoral students, exploring the influencing factors of anxiety among different types of doctoral students. It analyzes the mechanisms of these factors, addressing research issues, such as the assessment of anxiety levels, analysis of individual characteristic differences, and exploration of causes. The study expands the scope of influence path analysis in the field of research on doctoral students’ anxiety, helping them maintain a positive mindset to cope with academic research, thereby improving the learning efficiency and quality of life, and promoting high-level career development and personal growth. Based on the comprehensive analysis, the following conclusions were drawn, forming an intervention mechanism framework for doctoral students’ anxiety, as shown in Figure 7.

First, anxiety is prevalent among doctoral students. The research shows that over 40% of doctoral students experience anxiety, consistent with the findings at different times and in various countries (Liu et al., 2019; Muro et al., 2022; Singh, 2020). The high level of anxiety among doctoral students poses a serious threat to the successful pursuit of their degrees and their long-term development. Second, there are individual characteristic differences in the anxiety levels of doctoral students. Questionnaire analysis results indicate significant differences in anxiety levels based on gender, age, grade, marital status, and meeting frequency. Doctoral students at different academic densities, developmental stages, and living conditions exhibit diverse psychological states and behavioral choices in planning development expectations, addressing research challenges, and balancing study time, leading to varied academic and emotional experiences. The cognitive and actual adaptation levels determine the different anxiety levels among doctoral students under varying objective conditions. Third, both internal psychological factors and external environmental factors jointly contribute to the anxiety of doctoral students. Combining in-depth interview analysis with existing academic research, various factors influence the anxiety levels of doctoral students from multiple dimensions. The graduation orientation determines the difficulty and challenges of academic investment for doctoral students. Full-time doctoral students develop anxiety under high graduation standards, while part-time doctoral students experience academic progress anxiety due to a lack of resource support, a disconnection from academic culture, and inconvenient advisor guidance. Employment orientation faces intensified market competition, leading to career development uncertainty and anxiety among full-time doctoral students. Marital pressure affects the future life stability of doctoral students. Individual factors reflect the conflict between psychological expectations and real conditions. Full-time doctoral students’ pursuit is influenced by family conditions, while part-time doctoral students develop anxiety due to time conflicts and divided attention while balancing study, work, and family responsibilities.

Based on this, to effectively address the anxiety issues faced by doctoral students, the following targeted recommendations are proposed. At the societal level, establish a mechanism for resource integration and sharing. First, construct a social support network for psychological health. This involves aggregating psychological counseling institutions and volunteers from various sectors, increasing the awareness of doctoral students’ anxiety, and coordinating specialized psychological health service platforms for different types of doctoral students to enable precise intervention. Second, facilitate doctoral student employment and talent exchange channels. This involves providing career guidance tailored to the market needs for different types of doctoral students. Third, organize social activities for doctoral students. This focuses on addressing their relationship needs and expanding their social circles. At the university level, improve psychological health guidance and support services. The first is to optimize the support system for the development of doctoral students’ health, to carry out regular monitoring of doctoral students’ anxiety, to track changes in the anxiety level of doctoral students, and to identify problems and intervene in a timely manner; the second is to strengthen non-academic support services for doctoral students’ anxiety, to optimize online self-help resources, and to offer seminars to teach students strategies to maintain their psychological health (e.g., Positive Mindfulness Training) (Waight & Giordano, 2018). Trait mindfulness can facilitate individuals in developing autonomous behaviors that align with their needs and goals, thereby aiding them in maintaining a state of vitality (Hu et al., 2024); the third is to establish an industry–academia–research-based employment chain for doctoral students and offer employment guidance courses to help doctoral students clarify employment trends and improve their own employment competitiveness; the fourth is to establish a peer-learning system to strengthen doctoral students’ scientific research ability through knowledge exchange and skill complementation, enhance their self-identity, and reduce physical and mental stress. At the supervisor level, deepen communication and collaboration between teachers and students. Supervisors should advocate for a healthy, open, and inclusive academic atmosphere, take into account the cultivation plan, students‘ needs, and their ability level, respect students’ individual differences, formulate targeted development plans for doctoral students, strengthen scientific research discussions and psychological communication with part-time doctoral students, understand their academic needs and life status, and alleviate their anxiety. At the level of doctoral students, maintain a healthy lifestyle. A series of studies has shown that there is a two-by-two positive correlation between the stress level, anxiety level, and depression level of doctoral students (Marais et al., 2018; Reay, 2018; Singh, 2020), and all three often appear simultaneously in the abnormal psychological responses of doctoral students, who should, by means of physical exercise, participate in social activities and contacting friends and family, etc.; doctoral students should strengthen the interaction with external groups and environments to alleviate the sense of loneliness and stress in the process of scientific research, so as to diminish the possibility of their anxiety state and depression.

## 6. Limitation

From the perspective of research objects, our study, which took doctoral students currently enrolled in China as the representative sample, examined the anxiety states of doctoral candidates within a unique cultural context. However, due to the differences in the social environment, education system, and values, our research cannot comprehensively represent the anxiety level of all doctoral students in the world. On this basis, a broader sample can be included in the future to explore the research on the psychological health status of doctoral students in the world.

From the perspective of the research methods, firstly, in order to assess the anxiety status of on-campus doctoral students quickly and effectively, we adopted the GAD-7 scale as the assessment tool to enhance the reliability of the research. However, when it comes to the specific group of doctoral students, the GAD-7 scale has difficulty comprehensively covering the anxiety related to specific situations, such as course pressure, research pressure, and career prospects, and cannot sensitively and precisely reflect the unique anxiety symptoms of doctoral students. Future studies should fully take this limitation into account and conduct a comprehensive assessment of doctoral students’ anxiety by combining other relevant methods. Secondly, while this study has leveraged qualitative research methodologies to gain an in-depth comprehension of the individual experiences, contextual nuances, and underlying meanings associated with doctoral students’ anxiety, it is imperative to acknowledge the inherent limitation of reflexivity that arises within this research paradigm. The process of qualitative research, which is fundamentally underpinned by effective interactions between the researcher and the researched, is inherently susceptible to the unconscious infiltration of the researcher’s personal positions, viewpoints, experiences, and emotional biases. Consequently, this may inadvertently influence the interpretation and understanding of the anxiety psychological phenomena experienced by doctoral students, thereby potentially compromising the precision and objectivity of the research findings. Thirdly, we have adopted the cross-sectional data-collection method, which can instantly capture the current anxiety state of doctoral students in school, but it is limited by the difficulty in summarizing the dynamic characteristics of the anxiety state over time, which cannot fully explain the complex and changeable nature behind the anxiety. Future research endeavors could encompass several rounds of data collection to track the psychological states of doctoral students at various intervals, thereby observing the evolving trends in their anxiety levels over time.

From the perspective of the research content, as the analysis of the mechanism of anxiety of doctoral students in this study focuses on identifying the differences in the individual characteristics of doctoral students and exploring the paths of the influencing factors, it does not include all the multidimensional influencing factors in the framework of the quantitative analysis, which makes it difficult for us to accurately obtain the effects of psychological and environmental factors other than demographic characteristics on the psychology of doctoral students based on a large number of samples, and we are unable to clarify the interaction relationship among the elements within the conceptual framework of the influencing factors. Future research can deepen the quantitative analysis means and process on the basis of the conceptual framework of influencing factors and combine the path and relationship of influencing factors, so as to improve the quantitative analysis of the influencing mechanism of anxiety in doctoral students.

## Figures and Tables

**Table 2:** The basic information distribution of subjects (N = 638).

Variable	Character	Number	Percent (%)
Gender	Male	402	63.0
	Female	236	37.0
Age	≤25	102	16.0
	26–29	397	62.2
	30–34	81	12.7
	35–39	50	7.8
	≥40	8	1.3
Way of studying	Full-time	552	86.6
	Part-time	86	13.4
Grade	First-year	143	22.4
	Second-year	121	19.1
	Third-year	141	22.1
	Fourth-year	145	22.7
	Fifth-year	59	9.2
	Sixth-year	29	4.5

**Table 3:** Basic information of interviewed doctoral students.

Code	Gender	Age	Grade	Major Category
S1	Male	26	First-year	Humanities and Social sciences
S2	Female	29	First-year	Psychology
S3	Male	25	Second-year	Engineering
S4	Male	28	Second-year	Psychology
S5	Female	30	Third-year	Humanities and Social sciences
S6	Female	29	Third-year	Humanities and Social sciences
S7	Female	32	Fourth-year	Engineering
S8	Male	33	Fourth-year	Psychology
S9	Female	30	Fifth-year	Humanities and Social sciences
S10	Female	29	Sixth-year	Engineering

## Data Availability

The data can be made on request. If necessary, please contact Bai Fan for the acquisition of raw data (13001025985@163.com).

## Notes

1 Data Sources: Survey of Earned Doctorates and Chinese Education Statistics (2011–2022).

2 Data Sources: Chinese Education Statistics (2012–2022).

## Appendix A

**Table A1:** Conceptual Categories Formed by Open Coding.

Conceptual Categories	Concept	Description
Tutor Tasks and Student Relationship	Tutor has many tasks	More Chinese-style mentors play the role of landlords, especially in disciplines like science, particularly in the four big pits of biochemistry and materials science. They like to use the word “push”. Almost all mentors are in a state of being pushed when they were students, and they continue to push students. (S4)
	The tutor is not available	His teaching method is relatively straightforward. He would point me to a research direction he thinks is worth exploring or could yield research results, without providing detailed guidance. I am required to collect the necessary information myself. Then, 2–3 months later, he would suddenly call me, asking for a simple first draft or an initial model. Throughout the process, he remains uninvolved. (S3)
	Tutor is dictatorial and forceful	I feel like the teacher expects me to work 24 h a day. Sometimes when I try to explain that I have other things to attend to, the teacher just doesn’t understand and insists on giving orders that I must follow. (S4)
Research Pressure	Heavy task	During my studies, I have been researching multiple topics simultaneously, and sometimes I can’t even distinguish them myself. (S7)
	Papers require a large number of journals with high grades	During my studies, I have been researching multiple topics simultaneously, and sometimes I can’t even distinguish them myself. (S5)
	Graduation thesis has many words and high requirements	During graduation season, we have to complete a thesis of over a hundred thousand words, undergo multiple rounds of revisions, submit it for blind review, and participate in many rounds of defense. Just thinking about it is overwhelming. (S3)
Graduation Procedures	Graduation process complex and tedious	From the qualification audit for doctoral studies, to the proposal defense, to the mid-term assessment, and then to the final defense, followed by blind review and evaluation of academic achievements over the four years, the process of submitting a series of materials is not as simple as it sounds. (S7)
	The years are long and the difficulties are great	Only when I actually pursued a Ph.D. did I realize how easy it is to extend the deadline. I had never thought this would happen to me before, and I feel very confused. (S9)
Employment Choices	There are few professional jobs	I feel like my major is very disappointing. Although I have a Ph.D., there are no suitable jobs in my field. I might have to compete for positions outside my field. (S1)
	Job options narrow	After obtaining a Ph.D., I feel that the scope of employment has become narrower. It seems that I can only do research, and I feel incapable of practical work that requires strong skills. (S2)
	Not interested in work	The jobs I can currently find are all things that I find annoying. Just thinking about them makes me feel uninterested and uninspired. (S3)
Employment Salary	Pay is not proportional to effort	I have been studying until I was 30 years old, spending a total of ten years on my bachelor’s, master’s, and Ph.D. Compared to others, I have invested more time, energy, and money. However, the salary I can get after graduation is not much higher than that of a master’s degree holder. I feel that I cannot accept this. What is the meaning of my educational investment then? (S5)
	The salary is not enough to support living in Beijing	Even if I get a Ph.D. and become an elite in everyone’s eyes, even if I have an annual salary of 500,000 RMB, if I want to buy a decent house in downtown Beijing, I would have to live without eating or drinking for 20 years. How can I establish myself? (S3)
Job-hunting disadvantage	Women are at a competitive disadvantage in the workplace	The world is too hostile to women. I just graduated with a Ph.D., and the organization assumes that I should be getting married and having children at my age. Other male students, who are not even half as competent as me, can get employed, but they don’t want me. (S10)
	Old age leads to employment difficulty	I find age quite terrifying. Nowadays, even fresh graduates have age restrictions for employment. Even for Ph.D. holders, some units only accept applicants up to the age of 35. For someone like me, who started studying late, this has a huge impact. (S8)
	The gap between expectation and reality is too large	I find age quite terrifying. Nowadays, even fresh graduates have age restrictions for employment. Even for Ph.D. holders, some units only accept applicants up to the age of 35. For someone like me, who started studying late, this has a huge impact. (S8)
Pressure of competition	The current pattern of employment is not good	In recent years, due to the impact of the COVID-19 pandemic, the overall economic environment in China has been poor. Many industries, such as education, are declining, and layoffs are happening everywhere, making it even harder to find a job. (S7)
	There is little demand for talents in advanced fields and great competitive pressure	When we Ph.D. graduates come out, we should go to some high-tech fields, but these positions are few and far between. Graduates from our major, even those from “985 universities”, are completely saturated. (S6)
Emotional experiences	No time for love	How can I have time for dating? I have countless tasks every day, and my advisor calls me in the middle of the night. I don’t even have time to eat, let alone dating! (S3)
	Inability to deal with intimate relationships and low emotional intelligence	I may have been studying all the time and have no hobbies. I don’t know how to chat with girls. I’ve had a few crushes, but I don’t know how to express them, so there are no stories. (S3)
Conceptive factors	Female doctor is the “third sex”	It’s said that there are three types of people in the world: men, women, and female Ph.D. holders. The general perception is that female Ph.D. holders cannot get married. (S10)
	Age anxiety caused by “leftover women”	Oh, not to mention 30 years old, now even after 25 years old, it’s considered the age of leftover women. I’m still studying, worrying about my thesis during the day and marriage at night. The older I get, the more anxious I become. (S5)
Spouse criterion	Soul friends are hard to find	When it comes to dating, I must find someone with similar values and interests. I hope the other person has the same hobbies as me, and we have a sense of “having missed each other” when we meet. I haven’t found that yet. (S2)
	Ability matches are hard to find	I’m already a Ph.D., and I expect the other person to be a Ph.D. too. I expect us to be socially and economically compatible. But I can’t meet such a person, and I’m only demanding others based on my own abilities. (S3)
	The material foundation is difficult to secure	To consider marriage, you must have financial capability, especially to establish yourself in Beijing, you need even more support. I’m still in the studying stage, with only some subsidies, which is not conducive to marriage. I may need to work harder for several more years. (S4)
Others’ expectations	Parental expectation	Since I started my Ph.D., my parents have always made me feel like I have to achieve great things. This makes me feel very guilty and under a lot of pressure. (S1)
	Mentor expectation	I also feel that maybe he had too high expectations when he accepted me. Maybe I’m just a process of constantly disappointing him, which mainly leads to disappointment in myself and a complete lack of confidence and confusion. (S5)
	Social expectation	The social expectations for Ph.D. holders are exaggerated, completely mythical. It seems that we are all changing the world, but I feel that I can’t live up to such an evaluation. (S8)
Peer pressure	Peer financial pressure	My friends who didn’t study as much as me can now support themselves financially, and some have even become big bosses. But I only have a meager subsidy, and it’s hard to even eat in the cafeteria. This makes me afraid to have hobbies and afraid to pursue girls. (S4)
	Peer pressure to get married	This year, I attended three classmates’ weddings. Seeing them so happy, some of them already have children, their lives have taken on more significant meaning, while I’m still studying. (S9)
Family status	The family of origin has financial difficulties	I’m different from my classmates. My family can’t provide me with financial support. I have a younger brother in school, and my parents are both retired. I have to find a way to earn more money to support my family. (S3)
	Family unsupport	My parents are not very supportive of my continued education. They hope I can focus on preparing for pregnancy, which makes me afraid to tell them anything. I feel like I’m facing a tougher situation with this choice. (S9)
Self-perception	Doctoral expectations are not consistent with the facts	My current life is completely different from what I expected when I chose to pursue a Ph.D. I never imagined this pace of life before. (S2)
	Decreased self-efficacy	After experiencing so many rejections in submitting papers, I now feel that my abilities are not suitable for a Ph.D. (S6)

## Appendix B

**Table A2:** Main categories of spindle code formation.

Main Category	Corresponding Category	Main Category	Corresponding Category
Graduation orientation	Tutor tasks and student relationship	Love and marriage pressure	Emotional experiences
	Research pressure		Conceptive factors
	Graduation procedures		Spouse criterion
Employment orientation	Employment choices	Individual factors	Others’ expectations
	Employment salary		Peer pressure
	Job-hunting disadvantage		Family status
	Pressure of competition		Self-perception
